# Comparative genomics of *Enterococcus* spp. isolated from bovine feces

**DOI:** 10.1186/s12866-017-0962-1

**Published:** 2017-03-08

**Authors:** Alicia G. Beukers, Rahat Zaheer, Noriko Goji, Kingsley K. Amoako, Alexandre V. Chaves, Michael P. Ward, Tim A. McAllister

**Affiliations:** 1 Faculty of Veterinary Science, School of Life and Environmental Sciences, The University of Sydney, Sydney, NSW Australia; 2 Lethbridge Research Centre, Agriculture and Agri-Food Canada, Lethbridge, AB Canada; 3 Canadian Food Inspection Agency, National Center for Animal Disease, Lethbridge Laboratory, Lethbridge, AB Canada

**Keywords:** Bovine feces, *Enterococcus*, Comparative genomics

## Abstract

**Background:**

*Enterococcus* is ubiquitous in nature and is a commensal of both the bovine and human gastrointestinal (GI) tract. It is also associated with clinical infections in humans. Subtherapeutic administration of antibiotics to cattle selects for antibiotic resistant enterococci in the bovine GI tract. Antibiotic resistance genes (ARGs) may be present in enterococci following antibiotic use in cattle. If located on mobile genetic elements (MGEs) their dissemination between *Enterococcus* species and to pathogenic bacteria may be promoted, reducing the efficacy of antibiotics.

**Results:**

We present a comparative genomic analysis of twenty-one *Enterococcus* spp. isolated from bovine feces including *Enterococcus hirae* (*n* = 10), *Enterococcus faecium* (*n* = 3), *Enterococcus villorum* (*n* = 2), *Enterococcus casseliflavus* (*n* = 2), *Enterococcus faecalis* (*n* = 1), *Enterococcus durans* (*n* = 1), *Enterococcus gallinarum* (*n* = 1) and *Enterococcus thailandicus* (*n* = 1). The analysis revealed *E. faecium* and *E. faecalis* from bovine feces share features with human clinical isolates, including virulence factors. The Tn*917* transposon conferring macrolide-lincosamide-streptogramin B resistance was identified in both *E. faecium* and *E. hirae*, suggesting dissemination of ARGs on MGEs may occur in the bovine GI tract. An *E. faecium* isolate was also identified with two integrative conjugative elements (ICEs) belonging to the Tn*916* family of ICE, Tn*916* and Tn*5801*, both conferring tetracycline resistance.

**Conclusions:**

This study confirms the presence of enterococci in the bovine GI tract possessing ARGs on MGEs, but the predominant species in cattle, *E. hirae* is not commonly associated with infections in humans. Analysis using additional complete genomes of *E. faecium* from the NCBI database demonstrated differential clustering of commensal and clinical isolates, suggesting that these strains may be specifically adapted to their respective environments.

**Electronic supplementary material:**

The online version of this article (doi:10.1186/s12866-017-0962-1) contains supplementary material, which is available to authorized users.

## Background

The genus *Enterococcus* is ubiquitous in nature and can be found in a range of habitats, being associated with soil, plants, fresh and salt water, sewage and the gastrointestinal (GI) tract of animals (including mammals, birds, fish, reptiles and insects) and humans [1]. Although typically a commensal of the human GI tract, enterococci are often associated with a variety of clinical infections including urinary tract infections, hepatobiliary sepsis, endocarditis, surgical wound infections, bacteraemia and neonatal sepsis [2, 3]. *Enterococcus faecalis* and *Enterococcus faecium* are the two species responsible for the majority of healthcare-associated enterococcal infections [4]. Difficulties in treating enterococcal infections have emerged due to their ability to readily acquire resistance to many antibiotics, most notably vancomycin. As a result, the ability to successfully treat clinical infections has been reduced [5].

Antibiotic use in livestock production has been correlated with the emergence of antibiotic resistant bacteria. This was first recognised in the 1990s when use of the glycopeptide avoparcin as a subtherapeutic growth promotant led to the emergence of glycopeptide-resistant *E. faecium* in livestock and poultry [6]. Consumption of meat products contaminated with resistant bacteria was suggested to lead to the transmission of glycopeptide-resistant *E. faecium* to healthy, non-hospitalised humans. This association demonstrated the transmission of resistant bacteria from animals to humans through the food chain [7, 8]. Consequently, avoparcin was banned as a growth promotant in Europe in 1997 [9]. However, many antibiotics continue to be administered subtherapeutically to livestock in North America. For example, tylosin phosphate, a member of the macrolide family, is administered subtherapeutically to cattle to control liver abscesses. We recently demonstrated that subtherapeutic administration of tylosin phosphate selected for macrolide resistant enterococci in the bovine GI tract [10]. Enterococci have the ability to transfer antibiotic resistance and virulence genes horizontally to other bacteria [11]. The creation of a reservoir of resistant enterococci in the bovine GI tract could promote the dissemination of antibiotic resistance genes (ARGs) to other bacteria, particularly if they are associated with mobile genetic elements (MGEs).

Comparative genomic analysis can be used to identify genes coding for virulence, antibiotic resistance and gene mobility as well as elucidate the evolutionary relationship among bacteria. The number of complete or draft genome sequences available for *E. faecalis* and *E. faecium* is 446 and 436, respectively, comprising the bulk of enterococcal genome sequences available (http://www.ncbi.nlm.nih.gov/genome), as several comparative genomic studies of these species have been conducted [12–14]. There are comparatively few draft genome sequences available for other *Enterococcus* spp. with only 11, 10, 6, 5, 2 and 1 genomes are available for *Enterococcus casseliflavus*, *Enterococcus hirae*, *Enterococcus durans*, *Enterococcus gallinarum*, *Enterococcus villorum* and *Enterococcus thailandicus*, respectively (http://www.ncbi.nlm.nih.gov/genome). Furthermore, there is a poor representation of genomic sequences for enterococci isolated from non-human sources [15].

Previously, we identified a number of enterococci from bovine feces that carried at least one ARG, but only a few isolates carried multiple ARGs [10]. We also identified *E. hirae* as the principle species of the bovine GI tract, with infrequent isolation of *E. faecium* and *E. faecalis*, the species associated with nosocomial infections in humans. In the current study, we selected twenty-one isolates of enterococci originating from bovine feces for whole-genome sequencing and comparative genomic analysis. We hypothesized that *E. faecium* and *E. faecalis* would present more genes coding for virulence and antibiotic resistance than other *Enterococcus* spp. isolated from bovine feces.

## Methods

### Isolate selection

Twenty-one *Enterococcus* spp. isolated from bovine feces including *E. hirae* (*n* = 10), *E. faecium* (*n* = 3), *E. villorum* (*n* = 2), *E. casseliflavus* (*n* = 2), *E. faecalis* (*n* = 1), *E. durans* (*n* = 1), *E. gallinarum* (*n* = 1) and *E. thailandicus* (*n* = 1) were selected for whole genome sequencing (Table 1). These were selected from an archive of isolates collected between 2004 and 2005, which were previously characterized by PFGE and antimicrobial susceptibility testing [10]. At least one representative of each species isolated from bovine feces was selected, and for *E. hirae* and *E. faecium*, selection was based on maximizing diversity as measured by PFGE profiles as well as selecting isolates that displayed unique antimicrobial resistance profiles.

**Table 1:** Genome characteristics of *Enterococcus* spp. isolated from bovine feces

Strain	No. contigs	Size (bp)	%GC	Genes	CDSs	ST^a^
*E. hirae* 1	32	2926392	36.7	2785	2712	–
*E. hirae* 2	29	2850950	36.7	2678	2631	–
*E. hirae* 3	81	3088947	36.6	2977	2906	–
*E. hirae* 4	28	3042973	36.7	2825	2753	–
*E. hirae* 5	28	2869170	36.8	2741	2670	–
*E. hirae* 6	62	2966815	36.6	2848	2777	–
*E. hirae* 7	235	2766361	37.0	2602	2535	–
*E. hirae* 8	47	2922437	36.7	2801	2730	–
*E. hirae* 9	47	3178271	36.6	2971	2899	–
*E. hirae* 10	71	3018341	36.6	2885	2814	–
*E. faecium* 11	111	2783595	37.9	2719	2648	214
*E. faecium* 12	182	2712126	38.3	2665	2597	Unknown
*E. faecium* 13	28	2772865	37.7	2659	2591	955
*E. thailandicus* 14	17	2603791	36.7	2495	2430	–
*E. villorum* 15	42	2994157	34.9	2834	2765	–
*E. villorum* 16	159	3056754	34.9	2907	2837	–
*E. faecalis* 17	34	2913318	37.3	2788	2729	242
*E. gallinarum* 18	41	3381991	40.5	3259	3197	–
*E. durans* 19	43	2931269	37.9	2723	2657	–
*E. casseliflavus* 20	85	3483586	42.6	3355	3295	–
*E. casseliflavus* 21	50	3639801	42.2	3436	3375	–

^a^ *ST* sequence type

### DNA extraction and sequencing

Genomic DNA was isolated using phenol:chloroform extraction. *Enterococcus* spp. were inoculated into 5 mL brain heart infusion (BHI; BD, Franklin Lakes, New Jersey, USA) broth and grown for 24 h in a shaking incubator (250 rpm; Excella E24 Incubator Shaker, New Brunswick Scientific) at 37 °C. To increase cell yield, 150 μL aliquots were inoculated into duplicate tubes containing 6 mL BHI (BD) and grown for 24 h as described above. Cells were harvested by centrifugation at 10,000 × *g* for 5 min into a 2 mL microfuge tube and stored at −20 °C until genomic DNA was extracted. For extraction, the pellet was thawed on ice and resuspended in 1 mL of sterile 0.85% NaCl to remove residual growth media. The cells were repelleted by centrifugation (10,000 × *g*) for 1 min and the supernatant decanted. The washed cell pellet was resuspended in 665 μL of T_10_E_25_ (10 mM Tris-HCl pH 7.5; 25 mM EDTA) and 35 μL of lysozyme (50 mg/mL; Sigma-Aldrich, Co., St. Louis, Mo, USA) was added. The tubes were incubated at 55 °C for 60 min as a pre-lysis step. A 175 μL of 5 M NaCl, 35 μL of proteinase K (10 mg/mL; Sigma-Aldrich) and 44 μL of 20% SDS were added to the suspension and mixed by gentle inversion before being incubated at 65 °C for 1–2 h until cell lysis was complete. The lysed cells were extracted once with phenol, once with phenol:chloroform:isoamylalcohol (25:24:1) and twice with chloroform. Ammonium acetate (10 M) was added to the mixture so as to achieve a final concentration of 0.5 M, followed by one volume of isopropanol to precipitate DNA. To encourage precipitation, the tubes were chilled on ice for 10 min before centrifuging at 10,000 × *g* for 10 min. The supernatant was decanted and the DNA pellet washed with 70% ethanol and allowed to air dry before dissolving in 400 μL of TE (10 mM Tris-HCl; 1 mM EDTA). RNase A was added to achieve a final concentration of 30 μg/mL and the mixture was incubated for 20 min at 37 °C. Duplicate solutions for each sample were pooled before performing a second extraction, once with phenol:chloroform:isoamylalcohol and once with chloroform. Ammonium acetate (10 M) was added to the final aqueous solution to achieve a final concentration of 2 M followed by one volume of isopropanol and chilled on ice for 10 min to precipitate DNA. The DNA was pelleted by centrifugation, washed with 70% ethanol, air-dried, dissolved in 100 μL of sterile deionized water and stored at −80 °C until genomic library construction.

Genomic library construction was performed using the Illumina Nextera XT DNA sample preparation kit (Illumina, Inc., CA, USA) following the manufacturer’s instructions and sequenced on an Illumina MiSeq platform (Illumina). High-quality reads were *de novo* assembled using SPAdes genome assembler version 3.6.0 software [16] and annotated using Prokka version 1.10 [17]. Multi-locus sequence typing (MLST) was performed using the MLST database (version 1.8) [18].

### Comparative analysis

Draft genome sequences of the 21 *Enterococcus* spp. were investigated for the presence of putative virulence genes and ARGs, mobile genetic elements (MGEs), bacteriophage, CRISPR-Cas and secondary metabolite biosynthetic gene clusters. Virulence genes were identified using VirulenceFinder (version 1.5) [19], and ARGs using a combination of ResFinder (version 2.1) [20] and the Comprehensive Antibiotic Resistance Database (CARDs) [21]. Results for ARGs were further verified using megaBLAST and hits were manually inspected. Genomes were investigated for integrative conjugative elements (ICEs) by homology searches using BLAST against 466 ICEs downloaded from the ICEberg database (version 1.0) [22]. To identify bacteriophage, the contigs of each draft genome were ordered based on alignment against a reference genome (see Additional file 1: Table S1) using progressive Mauve [23], and then analyzed for the presence of prophage using PHAST [24]. CRISPR-Cas were identified using the CRISPRdb [25] and secondary metabolite biosynthetic gene clusters using the Antibiotics and Secondary Metabolite Analysis Shell (antiSMASH) [26]. All alignments and BLAST searches were performed in Geneious version 9.0.4 (Biomatters, Ltd). Assignment of proteins into Clusters of Orthologous Groups (COGs) was performed using the Integrated Microbial Genomes (IMG) platform [27]. Blast atlases were generated by GView Java package software [28] using both alignment length and percent identity cut-off values at 80%. The phylogenetic analyses were conducted using a single nucleotide variant phylogenomics (SNVPhyl) pipeline [29]. Briefly the paired-end reads originating from Illumina sequencing of samples were aligned to the reference genome to generate read pileups (SMALT v.0.7.5; http://www.sanger.ac.uk/science/tools/smalt-0) followed by mapping quality filtering and coverage estimations. From the pileup, the variant calling, variant consolidation and single nucleotide variant (SNV) alignment generation of the final phylogeny was run through PhyML [30] using maximum likelihood. The resulting tree was visualized using FigTree software (http://tree.bio.ed.ac.uk/software/figtree/). When included in the analyses, complete genomes from NCBI database were run through Wombac Shred (https://github.com/tseemann/wombac/blob/master/bin/wombac-shred_fasta) or art illumina [31] to generate paired-end reads with 2 × 250 bp length and >30X coverage. The resulting reads along with the reads from experimental isolates and the reference genome were run through SNVPhyl pipeline to obtain phylogeny as described above.

## Results and discussion

### Sequencing statistics

A summary of the sequencing statistics for the 21 *Enterococcus* spp. genomes can be found in Table 1. The genomes ranged in size from 2.60 − 3.64 Mb with *E. thailandicus* exhibiting the smallest and *E. casseliflavus* the largest genome. There was considerable variation in the size of *E. hirae* genomes, suggesting large differences in the size of the chromosome between strains and/or the presence/absence of plasmids.

### Phylogeny

**Fig. 1:**
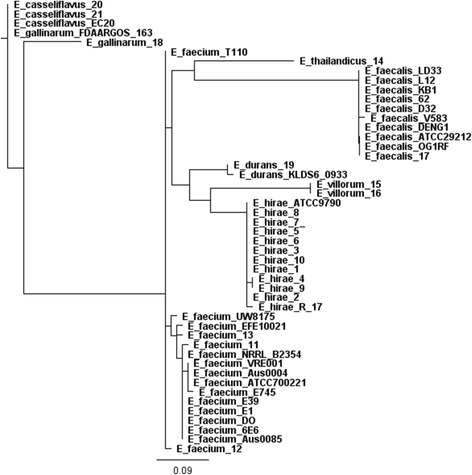
Phylogenetic tree constructed based on analysis of single-nucleotide polymorphisms (SNPs) of the core genes of 48 entercocci genomes, including the 21 isolates obtained from bovine feces in the present study. *Entercoccus faecalis*, *Entercoccus faecium*, *Enterococcus hirae*, *Entercoccus durans*, *Entercoccus casseliflavus* and *Entercoccus gallinarum* were compared using *E. faecium* strain T110 as a reference

Phylogenetic trees were constructed based on analysis of single-nucleotide variants (SNVs) of the core genes. A phylogenetic tree was constructed using all 21 sequenced *Enterococcus* spp. genomes as well as 27 complete enterococci genomes downloaded from the NCBI database (Fig. 1). The 27 compete genomes from NCBI included: *E. hirae* (2 strains; ATCC 9790, R17), *E. faecium* (13 strains; Aus0004, Aus0085, T110, 6E6, VRE001, E1, E745, E39, UW8175, NRRL B2354, ATCC 700221, EFE10021), *E. faecalis* (9 strains; LD33, L12, KB1, 62, D32, V583, DENG1, OG1RF, ATCC 29212), *E. durans* (1 strain; KLDS6_0933), *E. gallinarum* (1 strain; FDAARGOS163), and *E. casseliflavus* (1 strain; EC20). A probiotic strain, *Enterococcus faecium* T110 was used as an outgroup reference (Fig. 1). The assembled tree was consistent with the PFGE profile dendrogram observed from our previous study [10]. As expected, clustering was observed for genomes of the same species further verifying the identity of each species based on previous *groES-EL* spacer speciation [10].

**Fig. 2:**
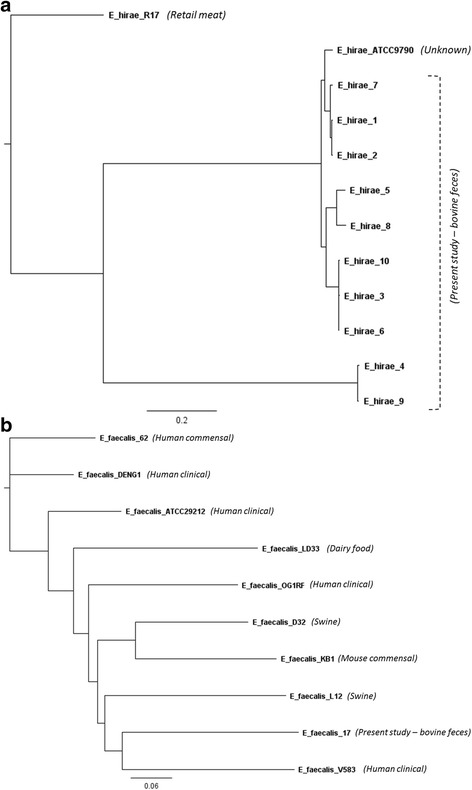
Phylogenetic trees of **a** *Entercoccus hirae* and **b** *Entercoccus faecalis* genome sequences from present study and complete genome sequences from the NCBI database based on analysis of single-nucleotide varients (SNVs) of the core genes. Origin of isolates are as indicated in the figures

Phylogenetic analyses were also conducted within species for *E. hirae*, *E. faecalis* and *E. faecium.* For *E. hirae*, as no genome from a clinical isolate was available and the origin of the type strain ATCC 9790 was unknown, *E. hirae* strain R17 (BioSample SAMN04892752) isolated from retail raw meat was used as an outgroup reference (Fig. 2a). Two distinct clades were identified with the majority of *E. hirae* constituting one clade and two genomes in the second (*E. hirae* 4 and *E. hirae* 9). The only *E. faecalis* isolate sequenced in this study, *E. faecalis*_17, clustered closely with a vancomycin resistant human clinical isolate, *E. faecalis* strain V583 (Fig. 2b). The other strains included in the analysis were of human-clinical, human-, swine- and mouse- commensal and dairy-related origin. Comparative phylogenetic studies with more *E. faecalis* isolates from bovine sources are required to understand the degree of relatedness between clinical and bovine isolates.

**Fig. 3:**
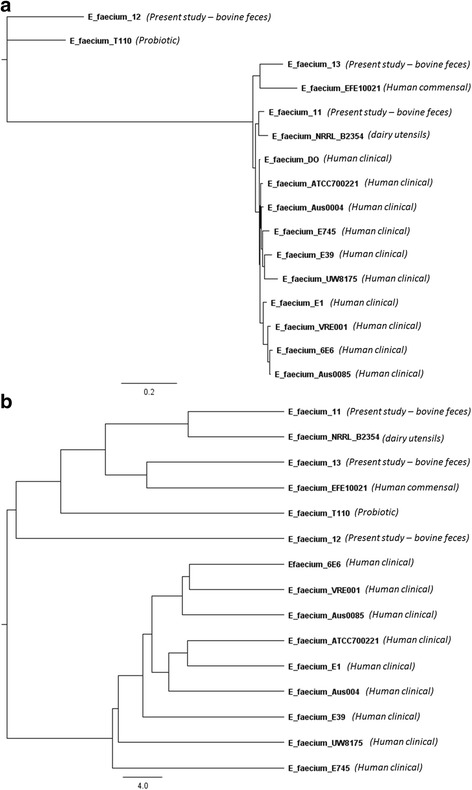
**a** Phylogenetic tree of *Entercoccus faecium* genome sequences from the present study and complete genome sequences from the NCBI database based on analysis of single-nucleotide varients (SNVs) of the core genes. **b** Relatedness tree of E. faecium genome sequences from present study and complete genome sequences from the NCBI database based on Pearson correlation similarity matrix analysis of accessory genes. Origin of isolates are as indicated in the figures

All three *E. faecium* genomes sequenced in this study were more closely related to NCBI genomes from commensal, probiotic and dairy isolates as compared to clinical isolates (Fig. 3a). To explore this further, we focused our phylogenetic analyses on the presence or absence of accessory genes, which further enhanced the distinction of these two clusters (Fig. 3b). Three complete NCBI *E. faecalis* genomes from non-clinical sources including human, swine, a probiotic and dairy clustered together with the bovine fecal isolates obtained in the current study. All other isolates included in the relatedness tree analysis were from various human clinical sources and generated a separate cluster. In the process of evolution, bacteria have accessorized their genomes with DNA from other bacteria with the help of mobile genetic elements (MGE) including plasmids, transposons, genomic islands and bacteriophages. The MGE-based accessory genome offers a very useful resource to bacteria for improving their fitness and adaptation within various environments, potentially through the development of pathogenicity and virulence. It is speculated that majority of *E. faecium* accessory genes contributing to the distinct clustering of clinical and commensal isolates in present study may be plasmid- or chromosomal MGE-borne. Further studies are required to identify those accessory genes to understand their role in enabling enterococci to adapt to specific environments.

### BLAST atlas

**Fig. 4:**
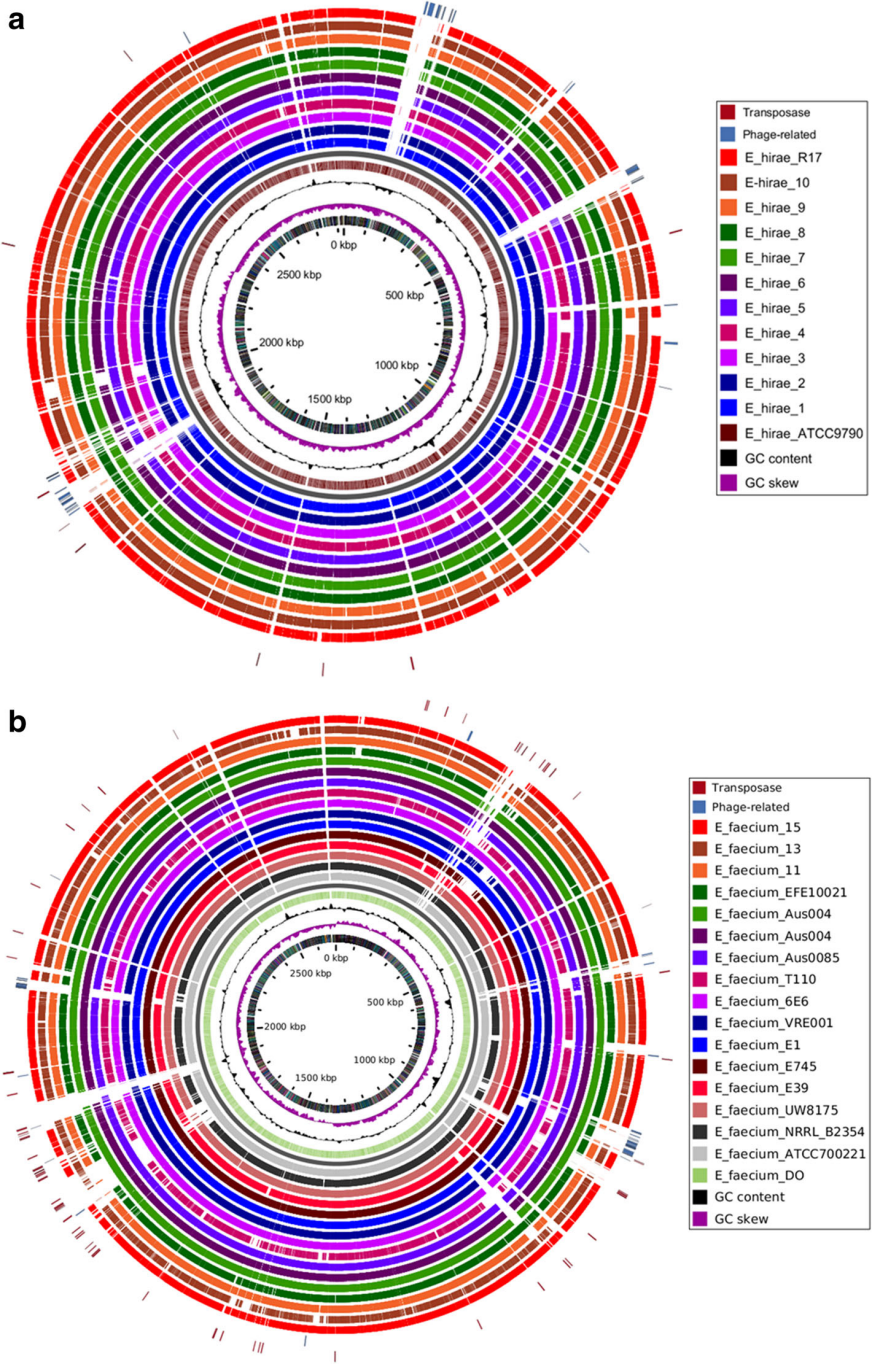
**a** Blast atlas of 10 *Enterococcus hirae* strains isolated from bovine feces and *E. hirae* strain R17 mapped against *E. hirae* ATCC9790. **b** Blast atlas of the genomes of 3 *Entercoccus faecium* isolates from bovine feces and 12 complete *E. faecium* genomes from the NCBI database mapped against reference sequence *E. faecium* DO. Blast atlases were generated by GView Java package software [28] using both alignment length and percent identity cut-off values of 80%. Based on the reference genomes, phage and transposon related regions/loci are indicated on the altas diagram

BLAST atlases were constructed using the genomes of isolated *E. hirae* and *E. faecium* and the reference genomes of *E. hirae* ATCC 9790 and *E. faecium* DO from the NCBI database (Fig. 4a & b). Of the *E. hirae* strains, *E. hirae* 7 exhibited the highest relatedness to the reference strain. *E. hirae* 7 and *E. hirae* 8 also shared phage-related genes with the reference strain (Fig. 4a). Several transposon-related loci were also shown to be shared with the reference genome. There were few variable regions identified among strains of *E. hirae*, illustrating their similarity in gene content. Likewise, the gene content among strains of *E. faecium* was also highly similar. However, several phage and transposon related loci from the reference strain appeared to be absent in commensal strains but present in clinical strains (Fig. 4b). This observation further supports their distinct segregation into independent clades.

**Fig. 5:**
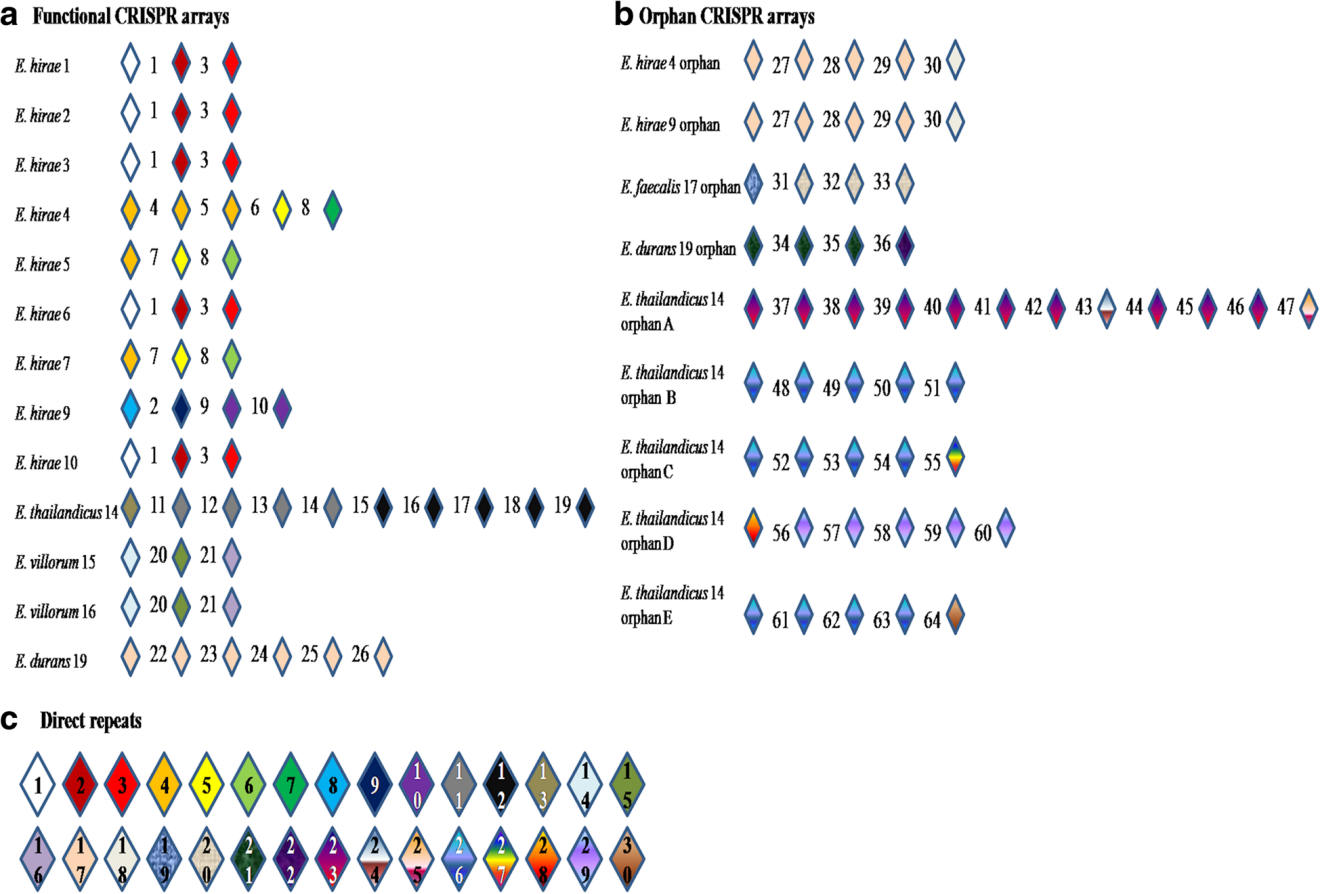
Schematic of CRISPR-Cas systems identified in whole genome sequence analysis of 21 *Enterococcus* spp. genomes. **a** Functional CRISPR array spacer and direct repeat organization. Diamonds represent direct repeats interspaced with numbers representing spacers. Spacer numbers correlate with sequences displayed in Additional file 1: Table S6. **b** Orphan CRISPR array spacer and direct repeat organization. Diamonds represent direct repeats interspaced with numbers representing spacers. Spacer numbers correlate with sequences displayed in Additional file 1: Table S6. **c** Numbered direct repeats. Numbers correlate with sequences displayed in Additional file 2: Table S5

### Clusters of Orthologous Groups (COGs)

Clusters of Orthologous Groups (COGs) are broad functional categories used to assign proteins related by function [32]. Functional categorization of proteins into different COGs (Additional file 2: Figure S1) revealed variation in the functional profile among *Enterococcus* spp., but the percentage of COGs assigned to cell cycle control, cell division, chromosome partitioning; extracellular structures; and intracellular trafficking, secretion and vesicular transport were similar among species. The percentage of COGs assigned to cell motility was greatest for *E. gallinarum* and *E. casseliflavus*, two species of *Enterococcus* that are known to be motile [12]. The percentage of COGs for cell motility was low for all other non-motile enterococci species [33]. There was little difference in the functional profile among strains of the same species with the exception of the mobilome: prophages, transposons category, in which inter-species variation was observed. Two *E. hirae* strains, *E. hirae* 4 and *E. hirae* 9, clustered together (Fig. 2a), two *E. faecium* strains (*E. faecium* 11 and *E. faecium* 12) and an *E. villorum*, *E. faecalis* and *E. casseliflavus* strain (*E. villorum* 16, *E. faecalis* 17 and *E. casseliflavus* 20, respectively) exhibited the greatest percentage of proteins assigned as phage and transposases.

Using the compare genomes function available in the IMG platform, we produced an abundance profile overview of the gene count for different COGs for all 21 *Enterococcus* spp. genomes (Additional file 1: Table S2). Van Schaik et al. [14] performed a COG-based functional comparison between *E. faecium* and *E. faecalis* in an effort to identify characteristics that distinguished the two species. In their analysis, they identified differences in sugar metabolism for the pentose sugar arabinose. They found COGs responsible for the metabolism (COG2160 and COG3957), uptake (COG4213 and COG4214) and degradation (COG3940) of arabinose to be present in *E. faecium* and absent in *E. faecalis*, attributing this to the inability of *E. faecalis* to metabolise arabinose [34]. Genes for these COGs, with the exception of COG4214 in *E. faecium* 12, were present in the *E. faecium* strains examined in this study and absent in our *E. faecalis* strain. Genes for these COGs were also present in *E. gallinarum* and *E. casseliflavus* strains, suggesting these species of *Enterococcus* also have the ability to metabolise arabinose (Additional file 1: Table S2). Ford et al. [35] previously documented that strains of *E. gallinarum* and *E. casseliflavus* that they examined were able to metabolise arabinose, but demonstrated poor growth compared to *E. faecium*. In the current study, *E. hirae*, *E. villorum*, *E. durans* and *E. thailandicus* all lacked genes for these COGs suggesting that they lacked the ability to metabolise arabinose, an outcome that has been biochemically confirmed by others [36–38]. Arabinose is a subunit of the plant polysaccharide hemicellulose and therefore would be in abundance in the GI tract of cattle [14]. Despite *E. faecium* being able to utilize arabinose as an energy source, this trait does not appear to provide a competitive advantage for this species to proliferate in the GI tract of cattle, considering *E. hirae* is the predominant species identified [10].

Van Schaik et al. [14] investigated other COGs involved in the metabolism of carbon sources from plants including COG4677, which is predicted to be involved in the metabolism of pectin, and COG3479, which is involved in the breakdown of coumaric acid and other components of lignocellulose. In our study, COG4677 was present in *E. faecium*, *E. durans* and *E. casseliflavus* and absent from *E. hirae*, *E. thailandicus*, *E. villorum*, *E. faecalis* and *E. gallinarum*, whilst COG3479 was present in *E. hirae*, *E. faecium*, *E. villorum* and *E. durans* and absent from *E. faecalis*, *E. thailandicus*, *E. gallinarum* and *E. casseliflavus*. These authors also highlighted a number of COGs present in *E. faecalis* that were absent in *E. faecium* including COGs for the utilization of ethanolamine as a carbon source. In the current study, *E. faecalis* possessed COGs for the utilization of ethanolamine, which were confirmed to be absent in *E. faecium*. Ethanolamine utilization has been demonstrated for *E. faecalis* [39], but not for other *Enterococcus* species. In the current study, these COGs were also identified in *E. gallinarum* suggesting this *Enterococcus* species may also utilize ethanolamine as an energy source, but to our knowledge this has not been biochemically confirmed. It is clear that different *Enterococcus* spp. have the ability to utilize various carbon sources allowing them to inhabit and survive in many diverse environments, including the GI tract of cattle. From this study, it was not apparent if *E. hirae* possessed specific traits for carbohydrate metabolism that may promote its abundance in the GI tract of cattle over other *Enterococcus* spp.

Van Schaik et al. [14] also investigated proteins involved in protection against oxidative stress. They identified the enzyme catalase (COG0753) was present in *E. faecalis* and absent in *E. faecium*. Examination of the different *Enterococcus* spp. in this study confirmed catalase to be specific for *E. faecalis* as it was absent from all other species. In the presence of heme, *E. faecalis* exhibits catalase activity [40]. Catalase production has been speculated to play a role in virulence in pathogenic bacteria including *Staphylococcus aureus* [41, 42]. *E. faecalis* can be exposed to oxidative stress as part of the host defence against invasion [40]. Catalase production may offer some protection against oxidation during invasion, contributing to the virulence of *E. faecalis*. Other mechanisms in *E. faecium* may play a role in the oxidative stress response, including the production of glutathione peroxidase (COG0386) [14]. With the exception of *E. faecalis,* this COG was present in all species of *Enterococcus* examined in this study, demonstrating the different strategies *Enterococcus* spp. use to combat oxidative stress*.*

### Multi-locus sequence typing (MLST)

Multi-locus sequence typing (MLST) has been used to study the population structure and evolution of *E. faecium* and *E. faecalis* [43, 44]. This technique involves sequencing and analysis of housekeeping genes and assignment of a sequence type (ST) [44, 45]. In the current study *E. faecium* 11, *E. faecium* 12 and *E. faecium* 13 were classified as ST214, unknown and ST955, respectively, and *E. faecalis* 17 as ST242 (Table 1). The lack of an assignment of a ST for *E. faecium* 12 suggests there are STs that have yet to be defined within the MLST database. STs can be assigned to a clonal complex (CC) based on their similarity to a central alleic profile [46]. MLST analysis of the population structure of *E. faecium* has identified that the majority of strains associated with nosocomial infections belong to the Clonal Complex 17 (CC17) [43]. For *E. faecalis* it appears that two complexes, CC2 and CC9, represent hospital-derived strains [44, 47]. The STs assigned to *E. faecium* and *E. faecalis* identified in the current study have been described previously [47–50] and are not associated with complexes of hospital-derived strains. There is currently no typing scheme available for other *Enterococcus* spp.

### Virulence genes

Virulence genes contribute to the pathogenicity of an organism. In this study, virulence genes were only detected in *E. faecium* and *E. faecalis*. All three *E. faecium* strains contained the *efaA* and *acm* genes, whilst *E. faecalis* contained a number of virulence genes including *efaA*, *ace*, *ebp* pili genes, *gelE* and *fsrB*. The *acm* and *ace* genes described in *E. faecium* and *E. faecalis*, respectively, are important for facilitating cell wall adhesion to host tissues [51, 52]. The *efaA* gene found in both *E. faecalis* and *E. faecium* also plays a role in adherence to host tissues and is a virulence factor involved in endocarditis [53, 54]*.* The *ebp* pili genes described in *E. faecalis*, comprising of *ebpA, ebpB* and *ebpC*, assist in adherence and biofilm formation [55]. The *gelE* gene in *E. faecalis* encodes for gelatinase, which hydrolyses gelatin, collagen, casein and haemoglobin [56]. Its expression is regulated by the two-component *fsr* system, with both *gelE* and *fsr* genes important in biofilm formation [57–59]*.*

In addition to these virulence genes, a number of bacterial sex pheromone genes were also present in *E. faecalis* including *cad*, *camE, cCF10* and *cOB1*. Certain conjugative plasmids found in *E. faecalis* respond to the secretion of bacterial sex pheromone genes from plasmid-free enterococci, inducing their transfer [60]. Sex pheromone response plasmids have rarely been described in other *Enterococcus* spp, but a few have been reported for *E. faecium* [61, 62]. The bacterial sex pheromones detected in the *E. faecalis* genome target the sex pheromone plasmids pAD1, pAM373, pCF10 and pOB1, respectively. Some of these plasmids encode features that can contribute to virulence such as pAD1 and pOB1, both encoding for a bacteriocin and hemolysin, and pCF10, encoding tetracycline resistance [63]. The pheromone cAD1 precursor lipoprotein *cad* gene was detected in all of the *Enterococcus* spp. isolates sequenced in this study, with 98–59% amino acid identities to *E. faecalis* strain FA2-2. Presence of the cAD1 precursor lipoprotein in these *Enterococcus* spp. increases their potential of receiving the highly conjugative pheromone-responding plasmid pAD1. The hemolysin/bacteriocin (cytolysin) encoded by this plasmid has been shown to contribute to virulence in animal models [64]. Therefore acquisition of this plasmid by these *Enterococcus* spp. could increase their virulence. Further analysis is required to determine if this sex pheromone precursor is able to induce transfer of pAD1 to *Enterococcus* spp. other than *E. faecalis*.

Virulence genes have mostly been characterized in *E. faecalis* and *E. faecium*, with little information available on the nature of these genes in other enterococcal species. A whole cytolysin operon has been reported in *E. durans* and cytolysin genes have been identified in dairy-associated *E. hirae* and *E. gallinarum.* Other virulence genes were also commonly detected in *E. durans*, such as the *esp* gene which is important for adhesion [65]. With the exception of *E. faecalis* and *E. faecium*, virulence genes were not detected in the sequence of the other *Enterococcus* isolates from the bovine GI tract. In addition to human clinical enterococci, virulence genes have been identified in enterococci from non-clinical environments [65–67] with *E. faecalis* having a greater prevalence of virulence genes than *E. faecium* [68, 69], an observation that aligns with our study.

### Antibiotic resistance genes

Enterococci can exhibit resistance to a number of antibiotics, partly due to their innate resistance to many commonly used antibiotics such as penicillin, but also due to their ability to successfully acquire resistance through horizontal exchange of ARGs on MGEs [70]. In this study we screened the 21 *Enterococcus* genomes against the ResFinder and CARDs databases for resistance genes (Table 2). Genes conferring resistance to vancomycin were only found in the genomes of *E. gallinarum* and *E. casseliflavus*, where the *van*C operon was present. The *van*C operon is intrinsic to these species of *Enterococcus* and provides resistance to low concentrations of vancomycin [71, 72]. Of the isolates examined in this study, only *E. casseliflavus*_20 displayed phenotypic resistance to vancomycin (Additional file 1: Table S3). The intrinsic resistance of *E. casseliflavus* and *E. gallinarum* can provide protection to concentrations of vancomycin as high as 32 μg/mL [73]. Vancomycin was present at 30 μg in the disks used for susceptibility testing [10], a concentration that was sufficient to inhibit the growth of *E. gallinarum*_18 and *E. casseliflavus*_21 inspite of the *van*C operon in these isolates. However, *van*C in *E. casseliflavus*_20 provided adequate resistance to allow growth of this isolate in the presence of vancomycin. The lack of vancomycin resistance genes in *Enterococcus* isolated from bovine feces was not surprising as avoparcin, a glycopeptide related to vancomycin, has not been used in cattle in North America [74].

**Table 2:** Antibiotic resistance gene profile of *Enterococcus* spp. isolated from bovine feces. Values represent % pairwise identity

Resistance gene	aph(3′)-III	aac(6′)-Ii	aac(6′)-Iid	aac(6′)-Iih	ant(9) -Ia	adeC	erm(B)	msrC	lsa(A) ^a^	lsa(E)	lnu(B)	tet(L)	tet(M)	tet(O)	sat4	vanC operon ^b^
Strain																
*E. hirae* 1				99.5			100					100	96.5			
*E. hirae* 2				99.5			100									
*E. hirae* 3				100			100							93.0		
*E. hirae* 4				98.9			100							100		
*E. hirae* 5				100			99.5							91.4		
*E. hirae* 6				100												
*E. hirae* 7				99.5												
*E. hirae* 8				100												
*E. hirae* 9				98.9										96.9		
*E. hirae* 10				100										92.8		
*E. faecium* 11	100	100			100	99.9	100	98.9		98.9	99.9	100	95.1		99.0	
*E. faecium* 12		100				92.9		95.4								
*E. faecium* 13		99.8				99.8		99.3								
*E. thailandicus* 14																
*E. villorum* 15																
*E. villorum* 16							99.6					100				
*E. faecalis* 17									99.5							
*E. gallinarum* 18							98.6									present
*E. durans* 19			100													
*E. casseliflavus* 20																present
*E. casseliflavus* 21							98.6									present

^a^Intrinsic to *E. faecalis*

^b^ *van*C operon consists of *van*C, *van*R-C, *van*S-C, *van*XY-C and is intrinsic to *E.gallinarum* and *E. casseliflavus*; GenBank accession numbers for resistance genes: *aph(3′)-III* (M26832.1), *aac(6′)-Ii* (L12710.1), *aac(6′)-Iid* (AJ584701.2), *aac(6′)-Iih* (AJ584700.2), *ant(9)-Ia* (JQ861959.1), *adeC* (CP003583.1), *erm*(B) (U86375.1), *msrC* (AY004350.1), *lsa*(A) (AY225127.1), *lsa*(E) (JX560992.1), *lnu*(B) (AJ238249.1), *tet*(L) (M29725.1), *tet*(M) (EU182585.1), *tet*(O) (Y07780.1), *tet*(32) (AJ295238.3), sat4 (U01945.1), *vanC* operon *E. gallinarum* (AF162694.1), *vanC* operon *E. casseliflavus* (EU151753.1)

Resistance genes to macrolides were present in a number of *Enterococcus* genomes sequenced, a finding that coincides with the fact that cattle were administered tylosin phosphate in their diets [10]. *Erm*(B) confers resistance to macrolide-lincosamide-streptogramin B (MLS_B_) antibiotics and was found in isolates of *E. hirae*, *E. faecium*, *E. villorum*, *E. gallinarum* and *E. casseliflavus*. In contrast, *msrC*, a macrolide efflux pump, was only detected in *E. faecium* (Table 2). This is consistent with Portillo et al. [75] who described *erm*(B) as the predominant gene conferring resistance to erythromycin in *Enterococcus* spp. and *msrC* in *E. faecium*. The presence of these resistance genes corresponds with the phenotypic resistance observed in these isolates (Additional file 1: Table S3). Interestingly, *E. hirae*_6, *E. durans*_19 and *E. casseliflavus*_20 exhibited resistance to macrolides even though no resistance genes to macrolides matched those in either the ResFinder or CARDs databases.

We previously reported that the *E. thailandicus* isolate sequenced in this study exhibited intermediate resistance to erythromycin (Additional file 1: Table S3) [10, 76]. Although there were no obvious macrolide resistance genes present, there were a number of genes identified as having multidrug efflux functions which may have contributed to intermediate resistance to erythromycin [76]. There is also the possibility that this phenotype was as a result of an unknown gene that codes for erythromycin resistance.

Genes conferring resistance to high concentrations of aminoglycosides were not detected in any of the genomes. Susceptibility to high concentrations of aminoglycosides was confirmed by the lack of phenotypic resistance to gentamicin and streptomycin (Additional file 1: Table S3). Enterococci are intrinsically resistant to low concentrations of aminoglycosides which is conferred by the genes *aac(6′)-Ii*, *aac(6′)-Iid* and *aac(6′)-Iih* present in *E. faecium*, *E. durans* and *E. hirae*, respectively (Table 2) [77, 78].

Genes coding for tetracycline resistance were detected in a number of genomes, including *E. hirae*, *E. faecium* and *E. villorum* (Table 2). *Tet*(L) encodes for an efflux protein whilst *tet*(M) and *tet*(O) encode for ribosomal protection proteins [79]. Anderson et al. [80] found *tet*(O) was the most prevalent gene encoding for tetracycline resistance in enterococci isolated from cattle, a finding that agrees with ours. Anderson et al. [80] reported *E. hirae* as the predominant species isolated from cattle and *tet*(O) was only resistance determinant associated with *E. hirae* in the current study. Detection of *tet*(M) and *tet*(L) in other isolates is not unexpected as both genes are also frequently detected in enterococci from animals including poultry, pigs, dogs, cats, rabbits, badgers, wildcats and birds [81–83]. Disk susceptibility testing revealed isolates containing *tet*(M) were resistant to doxycycline whilst those containing *tet*(L) or *tet*(O) were susceptible (Additional file 1: Table S3). It is possible that isolates that are sensitive to doxycycline are susceptible to other members of the tetracycline family. In general, bacteria that are resistant to doxycycline are also resistant to tetracycline and oxytetracycline [84, 85].

Only a few of the selected genomes contained ARGs to two or more antibiotics. Of particular interest was *E. faecium*_11, which contained at least 11 ARGs as inferred from the analysis of genome sequences (Table 2), including those conferring aminoglycoside, MLS_B_, pleuromutilin, streptogramin A, tetracycline and streptothricin resistance.

### Mobile genetic elements

Mobile genetic elements (MGEs) play an important role in horizontal gene transfer (HGT) of ARGs within and between bacteria from human and/or animal hosts [86–88]. MGEs include plasmids, transposable elements, prophages and various genomic islands such as integrative and conjugative elements (ICEs) [89]. A number of MGEs have been described in enterococci including transposons, plasmids and bacteriophage [90].

The well-known Tn*3*-like transposon, Tn*917*, which is widely distributed in enterococci was identified in several of the sequenced genomes. Four *E. hirae* strains (*E. hirae* 1, *E. hirae* 2, *E. hirae* 3 and *E. hirae* 4) and one *E. faecium* strain (*E. faecium* 11) had high sequence homology (>95%) to the Tn*917* transposon, previously described in *E. faecalis* [91]. All of these strains exhibited erythromycin resistance (Additional file 1: Table S3) [10], conferred by the *erm*(B) resistance gene present in Tn*917*. Other distinguishing features of this transposon include a transposase (TnpA) and a resolvase (TnpR) involved in the replicative mode of transposition [92].

The *erm*(B) gene was present in a number of other genomes including *E. hirae*_5, *E. villorum*_16, *E. gallinarum*_18 and *E. casseliflavus*_21. However, it did not align with the Tn*917* transposon. In *E. hirae*_5, the *erm*(B) gene was found on a contig associated with chromosomal genes. The tetracycline resistance gene *tet*(O) was also found in the vicinity of *erm*(B). Based on sequence information, *erm*(B) in the other three genomes appeared to be plasmid mediated. In *E. villorum*_16, the *erm*(B) and *tet*(L) genes were found on contigs associated with a plasmid sequence from an *E. faecium* strain UW8175 (GenBank accession no. CP011830.1). In *E. gallinarum_*18 and *E. casseliflavus_*21, the *erm*(B) gene was found on contigs associated with the plasmid sequence of pRE25 from an *E. faecalis* (GenBank accession no. X92945.2).

The tetracycline resistance genes *tet*(L) and *tet*(M) found in *E. hirae_*1 were located on a contig which shared 21,418 identical bp with the 25,963 bp transposon Tn*6248* of *E. faecium* strain E506 (GenBank accession no. KP834592). The genes responsible for transposition (*tnpA*) and insertion and excision of Tn*6248* (*tndX*) were absent, as was the chloramphenicol acetyltransferase gene (*cat*). This same contig also appeared to be associated with a plasmid sequence in *E. hirae* strain R17 (GenBank accession no. CP015517.1), suggesting this remnant transposon may be on a plasmid.

Integrative conjugative elements (ICEs) are self-transmissible elements that contain modules for their maintenance, dissemination and regulation [93]. In major Gram-positive human pathogens (e.g., *Enterococcus* spp., *Staphylococcus* spp. and *Streptococcus* spp.), tetracycline resistance is known to arise from the acquisition of the Tn*916*-family ICE carrying the *tet*(M) gene. The gene synteny in this family of ICE is well conserved, but there are differences in integrase (*int*) and excisionase (*xis*) gene sequences, insertion site specificity, and host range among family members [94–96]. The Tn*916* ICE was originally identified as an 18-kb conjugative transposon in *E. faecalis* DS16 [97, 98]. Variants of some Tn9*16*-*tet*(M) members, including Tn*916*, Tn*5397*, Tn*6000* or Tn*5801*, are widely spread among several genera within the Firmicutes, suggesting widespread dissemination of these elements. Many Tn*916*-like ICEs have a broad host range and are responsible for dissemination of tetracycline resistance through *tet*(M) in Gram-positive bacteria associated with humans and animals [88, 98, 99]. Recently, almost identical Tn*5801*-like genomic islands have been identified in different Gram-positive bacterial species of pet (*Staphylococcus pseudintermedius*) and human (*E. faecalis*, *S. aureus*, *Staphylococcus agalactiae*) origin, suggesting a horizontal transfer of these elements [100]. In our study, two ICEs belonging to the Tn*916*-family were identified in *E. faecium*_11. These ICEs exhibited homology to Tn*916* and Tn*5801*, each harboring a *tet*(M) variant, and appeared to be located within the chromosome. In Group B *Streptococcus*, the vast majority of Tn*916* and Tn*5801* are inserted into the core genome [101]. Once inserted in the genome, it is thought that Tn*916* and Tn*5801* are retained, as they impose a minimal impact on the biological fitness of the host bacteria [88, 101, 102].

A gene cluster *aadE*– *sat4*–*aphA*-*3* encoding resistance to streptomycin, streptothricin and kanamycin, previously described in *E. faecium* [103], was also found in *E. faecium*_11 associated with plasmid related contigs. This gene cluster has also been described in Tn*5405* within *S. aureus* [104] and Tn*1545* from *Streptococcus pneumoniae* [105], suggesting that it is widespread among Gram-positive bacteria.

### Bacteriophages

Bacteriophage mediated transduction of antibiotic resistance has been demonstrated in enterococci [106], and potential virulence determinants have been identified in phage associated with *E. faecalis* [107]. Phage found in enterococci usually belong to the *Podoviridae*, *Siphoviridae* or *Myoviridae*, but others including *Inoviridae*, *Leviviridae*, *Guttaviridae* and *Fuselloviridae* have also been reported [108, 109].

All *Enterococcus* genomes sequenced contained at least one putative phage, ranging in size from 8.0 to 70.3 kb (Additional file 1: Table S4). A total of 37 intact prophages were identified across the 21 sequenced genomes. *E. hirae* and *E. faecium* contained one to three intact prophages, whereas *E. faecalis* and *E. gallinarum* each contained two intact prophages and *E. durans* contained one intact prophage. *E. villorum* and *E. casseliflavus* contained up to four intact prophages whilst no intact prophages were detected in *E. thailandicus*. The intact prophages detected were from the *Siphoviridae*, *Myoviridae* or *Podoviridae* families, with prophage from the *Siphoviridae* family being most prevalent across all species examined (Additional file 1: Table S4). Prophages of the *Phycodnaviridae* family were identified in *E. faecium* and *E. villorum*. Its status was intact for only one of the *E. faecium* strains whilst it was questionable or incomplete in others (Additional file 1: Table S4). To our knowledge, phage from the *Phycodnaviridae* family have yet to be described in enterococci species. However, their presence in the rumen microbiome has been reported following metagenomic analysis [110].

### CRISPR-Cas

Clustered regularly interspaced short palindromic repeats (CRISPR) and CRISPR-associated (Cas) genes are a type of adaptive immune response described in bacteria against invading genetic elements such as phage and plasmids [111]. A CRISPR locus includes a CRISPR array flanked by various *cas* genes, with the array comprised of short direct repeats alternating with short variable DNA sequences called ‘spacers’ [111]. Three types of CRISPR-Cas systems have been described, distinguished by the presence of different Cas genes namely *cas3* for type I, *cas9* for type II and *cas10* for type III [112]. Recently, two additional types have been proposed to this classification system that includes type IV and type V [111]. CRISPR-Cas systems typically described in enterococci are of the type II variety. However, a recent report identified a type I system in *Enterococcus cecorum* [113, 114].

All *E. hirae* strains contained CRISPR arrays, except for *E. hirae*_8. CRISPR arrays were also detected in *E. thailandicus*, *E. villorum* and *E. durans* (Additional file 1: Table S5). The CRISPR arrays from these genomes were flanked by Cas genes, consisting of *cas9*, *ca*s*1*, *cas2* and *csn2* with the exception of *E. villorum* which lacked the *csn2* gene. CRISPR arrays flanked by these four Cas genes are classified as a type II-A system and are predicted to be functional as indicated by the presence of the core Cas genes *cas1* and *cas2* [112, 115]. Following the same nomenclature, the CRISPR-Cas system identified in *E. villorum* would also be classified as a type-II system, but its subtype is unclear.

Multiple CRISPR arrays can often be detected in bacterial genomes. However, not all CRISPR arrays may be accompanied by Cas genes. It is predicted that these arrays lie dormant or that Cas genes from other similar arrays may be sufficient for their activity [116]*.* Orphan CRISPR arrays (without Cas genes) [114] were identified by the CRISPRdb in a number of genomes, including two *E. hirae* strains and in *E. thailandicus*, *E. faecalis* and *E. durans* (Additional file 1: Table S4). No functional CRISPR arrays were detected for *E. faecium*, *E. faecalis*, *E. gallinarum* or *E. casseliflavus*.

Comparison of CRISPR arrays flanked by Cas genes revealed unique arrays between *Enterococcus* species, but some arrays were shared among strains of the same species (Fig. 5). Amongst the nine *E. hirae* strains, only four unique arrays were present. The arrays identified in *E. villorum* were identical for both strains. The largest array was identified in *E. thailandicus*. Arrays identified in the sequenced *Enterococcus* genomes contained between three and ten direct repeat (DR) sequences, alternating with spacer sequences (Fig. 5; Additional file 1: Table S6). A total of 26 unique spacer sequences associated with functional CRISPR arrays and an additional 38 unique spacers associated with orphan CRISPR arrays were identified (Additional file 1: Table S7).

In enterococci, it is hypothesized that the absence of CRISPR-Cas systems is associated with increased antibiotic resistance in isolates of *E. faecium* and *E. faecalis* [117]. In this study, *E. faecium* 11 lacked CRISPR-Cas and harbored several antibiotic resistance genes, reflecting this association. Palmer and Gilmore [117] detected identities between CRISPR spacer sequences and sequences of known pheromone-responsive plasmids and phage, suggesting CRISPR-Cas systems provide defence against these invading genetic elements. The authors hypothesized that the absence of CRISPR-Cas systems resulted in a compromised genome defence, enabling the acquisition of ARGs on MGEs. Palmer and Gilmore [117] did not detect spacer sequences with identities to transposons and hypothesized CRISPR-Cas systems may not provide defence against transposons. Several *E. hirae* strains in the current study contained functional CRISPR-Cas systems and the *erm*(B) resistance gene on a Tn*3*-like transposon, supporting this theory.

Functional CRISPR arrays and intact prophage were identified in most of the genomes sequenced in this study, with the exception of *E. thailandicus*. It is not surprising that these genomes contained prophage, as bacteriophage have developed strategies to avoid CRISPR regulation through the development of anti-CRISPR systems to enable integration into the genome [118]. In the case of *E. thailandicus*, spacers identified in CRISPR arrays aligned to incomplete prophage sequences with 100% sequence similarity and may possibly explain the lack of intact prophage in this genome. Spacer 60 aligned with both regions 3 and 4 of *E. thailandicus* prophage whilst spacer 12 aligned with region 4. None of the remaining spacers identified in CRISPR arrays had any sequence similarity to identified prophage.

### Secondary metabolites

Bacteriocins are ribosomally synthesized antimicrobial peptides produced by Gram-positive and Gram-negative bacteria that have antimicrobial activity against closely related bacteria [119]. In Gram-positive bacteria, they are classified into three major classes. Class I consists of the heat stable, modified peptides or lantibiotics, Class II describes the heat stable, unmodified non-lantibioitics and Class III consists of large proteins that are heat unstable [119, 120]. It is believed the production of bacteriocins by bacteria provides a competitive advantage to their survival in certain ecological niches [121].

Putative lantibiotics were identified in *E. hirae*, *E. thailandicus* and *E. gallinarum* whilst none were predicted in *E. faecium*, *E. villorum*, *E. faecalis*, *E. durans* or *E. casseliflavus*. Putative class II bacteriocins were identified in seven *E. hirae* strains (*E. hirae_*3, *E. hirae*_4, *E. hirae*_5, *E. hirae*_6, *E. hirae_*8, *E. hirae_*9, *E. hirae_*10), two *E. faecium* strains (*E. faecium*_11, *E. faecium*_13), *E. thailandicus*, *E. villorum* and *E. durans*. A putative bacteriocin identified in *E. faecium*_11 and *E. faecium*_13 had an amino acid identity of 99% to Enterocin A (Genbank accession no. AAF44686.1). Enterocin A was first described in an *E. faecium* strain isolated from fermented Spanish sausage [122]. Enterocin A inhibits a broad spectrum of Gram-positive bacteria including species of *Clostridium*, *Propionibacterium*, *Listeria* and *Staphylococcus* [123].

Until recently, terpenes were mainly considered secondary metabolites associated with plants and fungi, and were described in prokaryotes in only a few instances. These compounds serve a number of purposes including acting as antibiotics, hormones, flavor or odor constituents and pigments [124]. Since the advent of genomic sequencing, a number of presumptive terpene synthase genes have been discovered in bacteria [125]. Putative terpenes were identified in all *E. hirae*, *E. villorum*, *E. gallinarum*, *E. durans* and *E. casseliflavus* genomes sequenced in this study. None were predicted in *E. faecium*, *E. thailandicus* and *E. faecalis* genomes. The role of terpenes in enterococci remains unclear.

## Conclusions

This study has provided valuable insight about genetic differences observed among *Enterococcus* spp. isolated from bovine feces. We hypothesized that enterococci originating from bovine feces would lack genes coding for virulence, but would contain MGEs that could promote the dissemination of ARGs. We confirmed the majority of *Enterococcus* spp. isolated from bovine feces lacked virulence traits. The virulence traits that were identified were primarily associated with *E. faecium* and *E. faecalis*. As *E. faecium* and *E. faecalis* are not the predominant species of the bovine GI tract, the risk of transmission to humans through contamination of food products is likely low. Of most concern perhaps is dissemination of ARGs on MGEs. We identified that both *E. faecium* and *E. hirae* contained the Tn*917* transposon conferring MLS_B_ resistance suggesting that transfer of ARGs may occur in the bovine GI tract between *Enterococcus* spp. We also identified two ICE of the Tn*916* family that conferred tetracycline resistance in one isolate of *E. faecium*. As only a small number of isolates were examined in this study it is possible that other enterococci may be present in the bovine GI tract with ICE harbouring ARGs. As the cost of genomic sequencing continues to decline, further investigation of ICE using whole genome sequencing will help determine if there are linkages between enterococci isolates from bovine, the surrounding environment and human clinical sources.

## Additional files

Additional file 1: Table S1. Reference sequences for contig ordering using progressive Mauve. **Table S2.** Clusters of Orthologous Groups (COGs) gene abundance profile overview for all 21 Enterococcus spp. isolated from bovine feces. **Table S3.** Raw antibiogram data from disk susceptibility testing conducted previously [10]. **Table S4.** Putative prophage detected in Enterococcus spp. isolated from bovine feces. **Table S5.** Presence and absence of CRISPR arrays and intact prophage in Enterococcus spp. isolated from bovine feces. **Table S6.** Direct repeat sequences of CRISPR arrays found in Enterococcus spp. isolated from bovine feces. **Table S7.** Spacer sequences of CRISPR arrays found in Enterococcus spp. isolated from bovine feces. (XLSX 466 kb)
Additional file 2: Figure S1. Organisation of protein coding genes by Clusters of Orthologous Groups (COGs) category. (PNG 731 kb)

## Abbreviations

GI: Gastointestinal
HGT: Horizontal gene transfer
ICE: Integrative congugative element
IMG: Integrated microbial genomes
MGEs: Mobile genetic elements
MLS_B_: Macrolide-lincosamide-streptogramin B
MLST: Multi-locus sequence typing
PFGE: Pulsed-field gel electophoresis
SNPs: Single-nucleotide polymorphisms
