# Supplementary figures and images for: Comparative genomics of Enterococcus spp. isolated from bovine feces
Source: BMC Microbiol. 2017 Mar 8;17:52. doi: 10.1186/s12866-017-0962-1 (PMC5341189; doi:10.1186/s12866-017-0962-1)

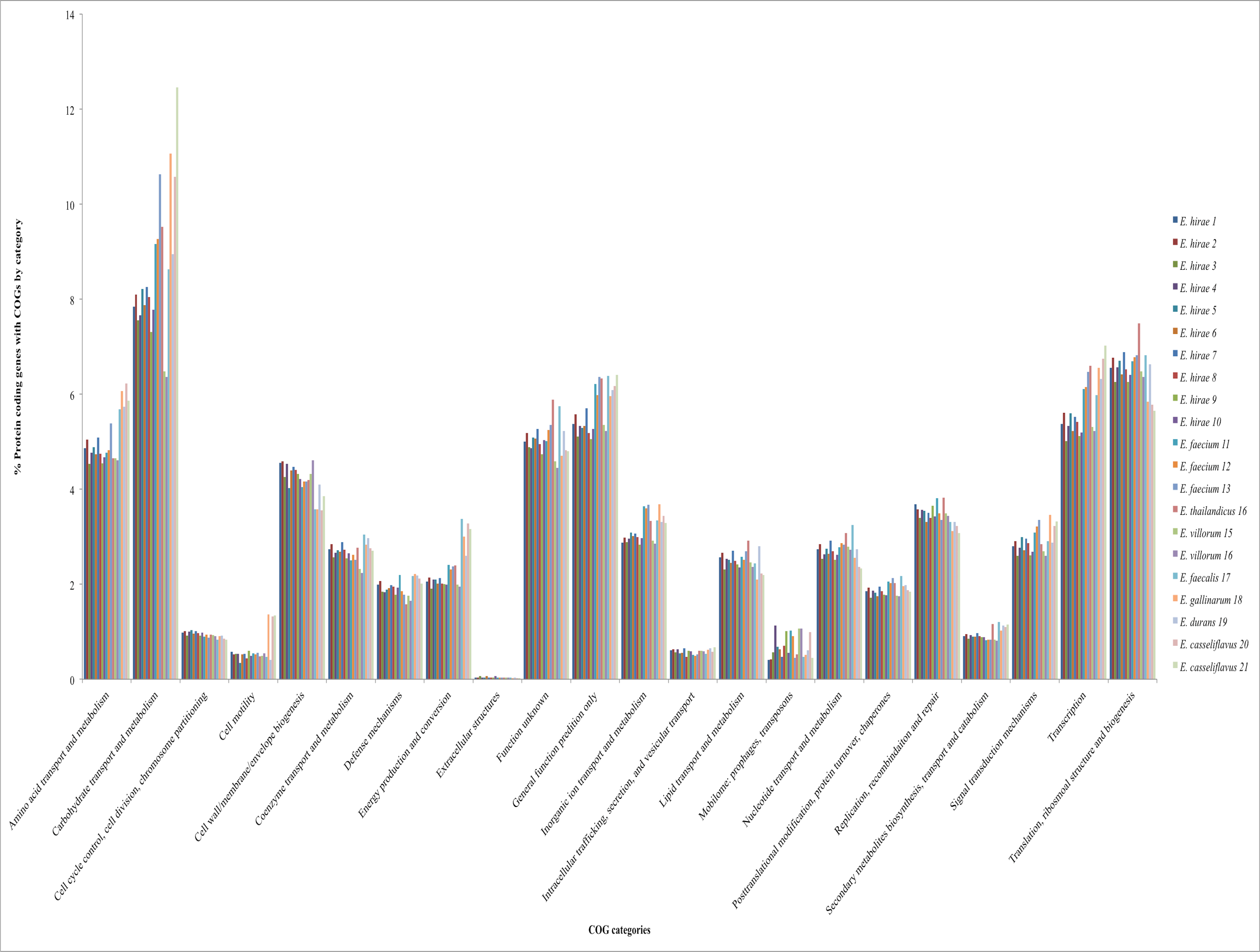

Supplement: Additional file 2: Figure S1. — Organisation of protein coding genes by Clusters of Orthologous Groups (COGs) category. (PNG 731 kb) [file 12866_2017_962_MOESM2_ESM.png]
